# New title: Re-evaluating environmental associations with meningitis risk across Africa

**DOI:** 10.12688/wellcomeopenres.24771.2

**Published:** 2026-02-13

**Authors:** Molly Cliff, Sally Jahn, Andre Bita Fouda, Anderson Latt, Clement Lingani, Caroline Trotter

**Affiliations:** 1 University of Cambridge Department of Veterinary Medicine, Cambridge, England, UK; 2 MRC Centre for Global Infectious Disease Analysis, Imperial College London, London, UK; 3 African Region, World Health Organization, Brazzaville, Brazzaville, Congo; 4 Regional Office for Africa, Emergency Preparedness and Response Cluster, World Health Organisation, Dakar, Senegal; 5 Inter-Country Support Team West Africa, World Health Organisation, Ouagadougou, Burkina Faso

**Keywords:** Bacterial meningitis, meningococcal meningitis, infectious disease epidemiology, epidemiology, climate change, public health

## Abstract

**Background:**

Previous analytical work, defining the distribution of meningitis epidemics in Africa is over 20 years old, with climate change representing an ongoing issue. We aim to update this analysis and determine if the meningitis belt geography and associated environmental risk factors have changed in the last two decades.

**Methods:**

Epidemic bacterial meningitis data from 2003–2022 were provided by WHO-AFRO. Districts across Africa were coded 1 if they experienced a meningitis outbreak and 0 if not. Monthly means of windspeed, rainfall, dust, and humidity were processed into climatic profiles using k-means clustering. We undertook logistic regression with meningitis epidemic history as the dependent variable and k-means clusters of rainfall, dust, humidity, and windspeed, alongside land-cover type as independent variables. A sensitivity analysis was conducted, excluding the Democratic Republic of Congo (DRC), due to limited laboratory confirmation of cases.

**Results:**

Rainfall, dust, windspeed and humidity demonstrated the strongest statistical association with outbreaks and were included in our final model. With a probability cut-off >0.4, our model had specificity and sensitivity of 82.07% and 82.22%, respectively, in identifying districts having experienced a meningitis epidemic. The Sahelian region had the highest risk of meningitis outbreaks (probability >0.8), consistent with previous findings. The inclusion/exclusion of the DRC had a significant impact on our model. In the full model the Republic of the Congo, Gabon, and Angola had a moderate risk of meningitis (probability >0.4), suggesting a possible expansion of the belt. However, when the DRC was excluded, no countries surrounding the meningitis belt were at risk for outbreaks, highlighting the importance of laboratory testing and case confirmation.

**Conclusions:**

The apparent extension of risk beyond the belt possibly reflects surveillance limitations rather than alterations in disease ecology. Where possible, laboratory confirmation should be used to support surveillance of suspected meningitis outbreaks and cases.

## Plain language summary

Bacterial meningitis is a significant public health issue within sub-Saharan Africa, where outbreaks show a distinct seasonal pattern. Cases peak within the dry season, characterised by low humidity and high dust levels. Most cases occur within 26 countries spanning Senegal to Ethiopia, known as the meningitis belt. In this study, we evaluated the statistical association between history of meningitis outbreaks across Africa and climatic factors including rainfall, dust, humidity, wind speed and land cover type. We aimed to see if there had been any changes in at-risk regions of meningitis in line with climate change.

This study revealed that the central and western part of the Sahelian region of Africa, with lower rainfall and humidity, had the highest risk of meningitis outbreaks. This includes countries such as Burkina Faso, Chad, Gambia, Niger and Nigeria. Several countries bordering the meningitis belt had a moderate outbreak risk, suggesting a potential expansion. However, when we excluded outbreaks from the Democratic Republic of Congo due to poor case confirmation, no countries surrounding the meningitis belt were at risk for outbreaks. It is important to improve diagnostic capacity within meningitis outbreaks, to accurately assess disease risk across the continent.

## Introduction

The African meningitis belt, which spans from Senegal, through to Ethiopia and Eritrea has a high seasonal incidence of bacterial meningitis ( WHO, 2025). The meningitis belt was first defined in 1963 by Lapeyssonie, as an area within sub-Saharan Africa including 11 countries with a high incidence of meningitis epidemics with approximately 300–1100 mm of annual rainfall ( Lapeyssonnie & World Health Organisation, 1963). It was found that meningitis outbreaks largely occured during the hotter dry season within sub-Saharan Africa, indicative of a correlation with seasonal weather patterns ( Lapeyssonnie & World Health Organisation, 1963). In 1971, an extension of the meningitis belt towards East Africa was proposed to include regions of Rwanda, Burundi, Uganda, Tanzania and Kenya based on observation of epidemics from 1911–1965 ( Mpairwe & Matovu, 1971). In 1987, Greenwood furthered this work to include areas of 17 countries, excluding the Democratic Republic of Congo (DRC) ( Greenwood, 1987). The meningitis belt currently spans 26 countries in sub-Saharan Africa. Globally, in 2019, approximately 50% of all meningitis cases in children under 5 years occurred within the meningitis belt ( GBD 2019 Meningitis Antimicrobial Resistance Collaborators, 2023).

Seasonality and climate variability have a key influence on the spatiotemporal distribution of bacterial meningitis across Africa. This influence is predominantly seen in meningitis cases caused by Neisseria meningitidis ( Greenwood, 1999). Within the dry season (December to April), driven by the onset of the Harmattan winds and increased dust concentration, disease incidence can reach 800 cases per 100,000 people ( Mohammed *et al.*, 2017). Bacterial meningitis incidence decreases and epidemics end with the beginning of the wet season characterized by pre-monsoon rainfall ( Agier *et al.*, 2013). Major bacterial meningitis outbreaks occur between every 5–12 years across the belt, where incidence can reach 1000 cases per 100,000 people ( Mohammed *et al.*, 2017).

Several climatic models have been able to highlight the spatiotemporal relationship between meningitis cases, and meteorological weather events ( Chen *et al.*, 2023; Tall *et al.*, 2022). A global generalized linear regression model found that increased temperature variability was significantly associated with higher meningitis risk ( Chen *et al.*, 2023). A modelling study across Burkina Faso found a significant association between meningitis incidence and both relative humidity and temperature ( Tall *et al.*, 2022). Several additional studies within the meningitis belt have concluded that humidity, dust, wind speed and rainfall are associated with meningitis epidemics ( Agier *et al.*, 2013; Besancenot *et al.*, 1997; Sultan *et al.*, 2005; Thomson *et al.*, 2006; Yaka *et al.*, 2008). However, these studies are often limited in their geographical scope to either one or a subset of countries within the meningitis belt. The specific mechanism is not known, but seasonal climatic factors may aid in the promotion of bacterial transmission and progression from carriage to invasive disease ( Mueller & Gessner, 2010). Work linking environmental factors and meningitis have been used successfully to support awareness, preparedness and response actions for meningitis outbreaks ( Dione *et al.*, 2022).

A 2002 study by Molesworth *et al.*, investigated the spatial distribution of meningitis epidemics in Africa by conducting a systematic review of district-level meningitis outbreaks in Africa between 1980 and 1999. This determined that in addition to those countries within the meningitis belt at the time Guinea-Bissau, Guinea, Côte d'Ivoire, Togo, the Central African Republic and Eritrea had a high epidemic burden ( Molesworth *et al.*, 2002). In 2003 this study was used to develop a logistic regression model investigating the relationship between epidemic location and environmental variables to locate districts within Africa with increased risk of meningitis epidemics. Zonal profiles of climatic variables were developed for use within the model. Absolute humidity and land-cover type were best able to predict meningitis epidemics, with dust and rainfall profiles also being independently associated with epidemic location ( Molesworth *et al.*, 2003). The association with dust is thought to be in line with the increase in dust following the Sahelian droughts of the 1970s and 1980s ( Palmgren, 2009). The model also demonstrated that climatic zones without distinctive seasonal changes including desert and forest areas are less likely to have epidemics as contrasted to regions with wet and dry seasons ( Molesworth *et al.*, 2003). A later evaluation of this risk model, examining meningitis outbreaks from 2000–2004 indicated that meningitis epidemics may be extending southwards within Togo, the Central African Republic, Cameroon, and Côte d’Ivoire ( Savory *et al.*, 2006). The authors concluded that the southwards extension of the meningitis belt was consistent with regional deforestation and desertification. It was suggested that alterations in land usage, combined with climatic factors, potentially led to a humidity reduction combined with an increase in dust, favouring epidemic conditions.

Our work will present an update on the associations between environmental variables and meningitis epidemic risk across Africa. Despite the changing epidemiology of bacterial meningitis since 2002 due to the introduction of Haemophilus influenzae type b, pneumococcal and particularly meningococcal vaccines, environmental risk factors for outbreaks remain consistent and relevant within the context of widespread vaccination. Historically, across the African meningitis belt, most bacterial meningitis outbreaks have been caused by meningococcal group A, previously responsible for 80–85% of all outbreaks ( WHO, 2025). Following the introduction of MenAfriVac from 2010 onwards, with initial campaigns taking place in Niger, Burkina Faso and Mali, there has been a significant reduction of meningococcal outbreaks within vaccinated countries ( Bender *et al*., 2023). It has been estimated that across the belt, the introduction of MenAfriVac led to a 57% decrease in the overall incidence of suspected meningitis cases, with this reaching a 91% reduction in Chad ( Trotter *et al*., 2017). By 2019 approximately 350 million people aged between 1–29 years within the meningitis belt had received a MenAfriVac dose as part of these campaigns ( Bender *et al*., 2023). In respect to routine immunisation, eight countries within the meningitis belt, mostly located in the Western Sahel, had a coverage of >60% as of 2021, with Burkina Faso having the highest routine immunisation coverage of 87.3% ( Bender *et al*., 2023).

However, whilst outbreaks of group A meningococcal (NmA) epidemics have decreased as a result there have been persistent outbreaks due to other meningococcal groups (NmW, NmX and NmC) as well as pneumococcal meningitis since 2013 ( Franklin *et al*., 2021; Ibarz-Pavón *et al*., 2011; Sidikou *et al*., 2016; Trotter *et al*., 2017). Furthermore, there have been several recent meningitis outbreaks across Togo, Nigeria and Niger since 2020, all of which have been caused by non-NmA bacterial pathogens or serotypes. Following on from the most recent meningococcal group C outbreak (NmC) in Nigeria, the country introduced the novel Men5CV vaccine, effective against serogroups A, C, W, X, and Y. From between 25th and 28th March 2024 a rollout of Men5CV across Nigeria, led to the vaccination of over one million individuals aged 1–29 ( Ukoaka *et al*., 2024). Although routine vaccination campaigns have altered the epidemiology of bacterial meningitis across the African continent, outbreaks still occur periodically, driven by both environmental and biological drivers.

It is important to understand the evolving impact of climate change on meningitis incidence, given that more than half of known human pathogenic diseases can be exacerbated by climate change ( Mora *et al.*, 2022). With 20 years having passed since the publication of Molesworth *et al.*’s logistic regression model, we aim to understand if the geography of the meningitis belt and risk factors associated with meningitis have changed. This paper serves to extend the temporal window of environmental associations with meningitis risk across the whole African continent by 20 years. By using 20 years of monthly meteorological data, our study focuses on climate dynamics, as opposed to shorter-term weather assessments. This puts our study ahead of Savory *et al.*’s 2006 paper, which only examined four years of data. Our longer-term analysis enables the isolation and identification of climatic trends amidst the natural variability of weather. Furthermore, since Molesworth *et al.*’s and Savory *et al.*’s publications, there have been significant advancements in the resolution of environmental meaning that updating previous work should serve to create a more reliable risk model. By repeating the methods used by Molesworth *et al.*, with new data, we are conducting a longitudinal epidemiological evaluation to look for a signal of change, before then going on to investigate the relationship between climate and meningitis further. This allows us to draw conclusions based on data without confounding our findings by changing methodology. Furthermore, most epidemiological studies of meningitis incidence across Africa, including Tall *et al*., focus on either one or a subset of belt countries. By focusing on the impact of bacterial meningitis across Africa, we can account for the differential effects of climatic factors across the continent, rather than examining localized atmospheric changes. Finally, this paper serves as an advancement to both Molesworth and Savory’s papers in that we reevaluated the environmental variables used within the final statistical model, considering the additional impact of both windspeed and temperature. This analysis is highly relevant to understanding the effect of climate on meningitis incidence in the context of a changing climate ( Cliff *et al.*, 2025).

## Methods

### Epidemiological data source

The World Health Organisation Regional Office for Africa (WHO-AFRO) provided us with data regarding meningitis epidemics reported to their enhanced meningitis surveillance system from 2003 through to 2022 ( Lingani *et al.*, 2015). In accordance with guidelines from the World Health Organisation (WHO) an epidemic was defined as any district reaching the epidemic threshold of 10 suspected meningitis cases per 100,000 population per week or with a seasonal cumulative incidence of 100 suspected meningitis cases per 100,000 population ( Trotter *et al.*, 2015).

The enhanced meningitis surveillance system reports disease data from 24 countries across sub-Saharan Africa which are at high risk of meningitis outbreaks (Extended data). This includes countries both inside and adjacent to the current meningitis belt. Whilst this excludes countries in North Africa that have been previously identified as high-risk, there have been limited outbreaks in that region during the study period ( Mazamay *et al.*, 2021). Outbreak metadata included the week, year, country and administrative level 2 district (ADMN2) of each meningitis epidemic as well as population size and number of cases.

As both pathogen and serotype data on individual outbreaks were unavailable, we did not examine the impact of vaccination as part of our main investigation, instead undertaking a sensitivity analysis excluding outbreaks that occurred after the introduction of MenAfriVac. For all countries across the meningitis belt, we used the date of MenAfriVac introduction from Bender *et al.* and set districts with outbreaks only occurring after vaccine introduction to zero. This allows us to understand how the introduction of MenAfriVac by country affects the association between climatic variables and bacterial meningitis incidence.

We did not consider vaccination as an independent variable within the model in part due to the static nature of the logistic regression model. Additionally, many outbreaks would have occurred before meningococcal and pneumococcal vaccinations were introduced, and the risk of epidemics from other serogroups/serotypes remains.

### Assigning outbreaks to ADMN2 districts

Meningitis epidemics were assigned to their respective ADMN2 districts using a district level shapefile of the whole continent of Africa. This was carried out using Stata/SE 18 with the shapefile being obtained from the Database of Global Administrative Areas (GADM). ADMN2 districts were used in all countries where available, apart from in Libya, Western Sahara and Comoros, where only ADMN1 level subdivisions exist. The GADM shapefile used within this study reflects ADMN2 district boundaries as of 2022.

However, district boundaries frequently change over time, making it more difficult to accurately assign older outbreaks to current administrative units. To address spatial mismatches and uncertainty with reported outbreak districts, we used an older WHO-provided ADMN2 shapefile of Africa to map historical epidemics to the 2022 GADM districts using R version 4.3.1.

For outbreaks that could only be assigned to a district with the older WHO shapefile we calculated spatial overlaps between the WHO and GADM ADMN2 shapefiles, to ensure temporal alignment. Districts with at least 50% spatial overlap were initially used to assign meningitis epidemics to ADMN2 districts within the GADM shapefile. To increase the number of epidemics, we were able to assign the process was repeated using a 30% overlap threshold. Alongside this, we carried out a short sensitivity analysis, changing the overlap thresholds to 60% and 40% to ensure that statistical modelling results demonstrate robustness to boundary uncertainty. All ADMN2 districts in the GADM continental shapefile were then assigned a binary value of either 1 or 0, representative of whether they had ever had a meningitis epidemic from 2003–2022.

### Environmental data sources and variables

Publicly available environmental data with uniform latitude-longitude grid-based coverage was used in this analysis. Variables used were originally derived from the interpolation of climatic anomalies across global weather station networks ( Table 1). All environmental data used was on a monthly level. When selecting environmental variables for inclusion, we both utilised variables identified in Molesworth *et al.*’s original analysis and examined the wider scientific literature for environmental variables with a known association with meningitis incidence.

**Table 1.** Characteristics of environmental variables to be included within risk modelling. We have included a comparison between resolution of environmental data used within this analysis to that used by Molesworth *et al.*

Variable	Time period	Resolution of grid squares	Units	Data source	Molesworth resolution
Average daily dew point temperature	2002–2020	0.1° x 0.1°	Degrees Kelvin (K)	Copernicus Climate Change Service, 2020	0.5° x 0.5°
Average daily surface pressure	2002–2020	0.1° x 0.1°	Hectopascals (hPa)	Copernicus Climate Change Service, 2020	0.5° x 0.5°
Average daily rainfall	2002–2022	5km	Monthly atmospheric precipitation (mm)	CHIRPS- ( Funk *et al.*, 2015)	0.5° x 0.5°
Average daily aerosol index (dust)	2002–2022 (excluding 2014–2016 due to data availability)	1.0° x 1.0°	Aerosol concentrations (unitless measure)	Copernicus Climate Change Service, 2019	1.0° x 1.0°
Land-cover type	2020	300m	Categorical variable numbered from 10-220	European Space Agency Climate Change Institute- ( Lamarche *et al.*, 2017)	1 km
Average monthly wind speed	2002–2022	4km	Wind speed (m/s)	Copernicus Climate Change Service- Hersbach *et al.* (2023)	Variable not included in Molesworth's 2003 analysis
Average monthly temperature	2003–2022	1.0° x 1.0°	Average temperature (K)	Goddard Earth Sciences Data and Information Services Center (2013)	Variable not included in Molesworth's 2003 analysis

From Molesworth’s original analysis we examined the impact of specific humidity (g/m ^3^), aerosol optical depth (unitless measure), rainfall (mm), and land coverage (categorical land cover class) on meningitis epidemics. We additionally examined the impact of temperature (K) and windspeed (m/s) as these variables are also known to have an association with meningitis epidemics across Africa. Details regarding the derivation of specific humidity, selection of environmental data sources and raster aggregation can be found within the extended data.

### Processing of climatic data

**Figure 1.**
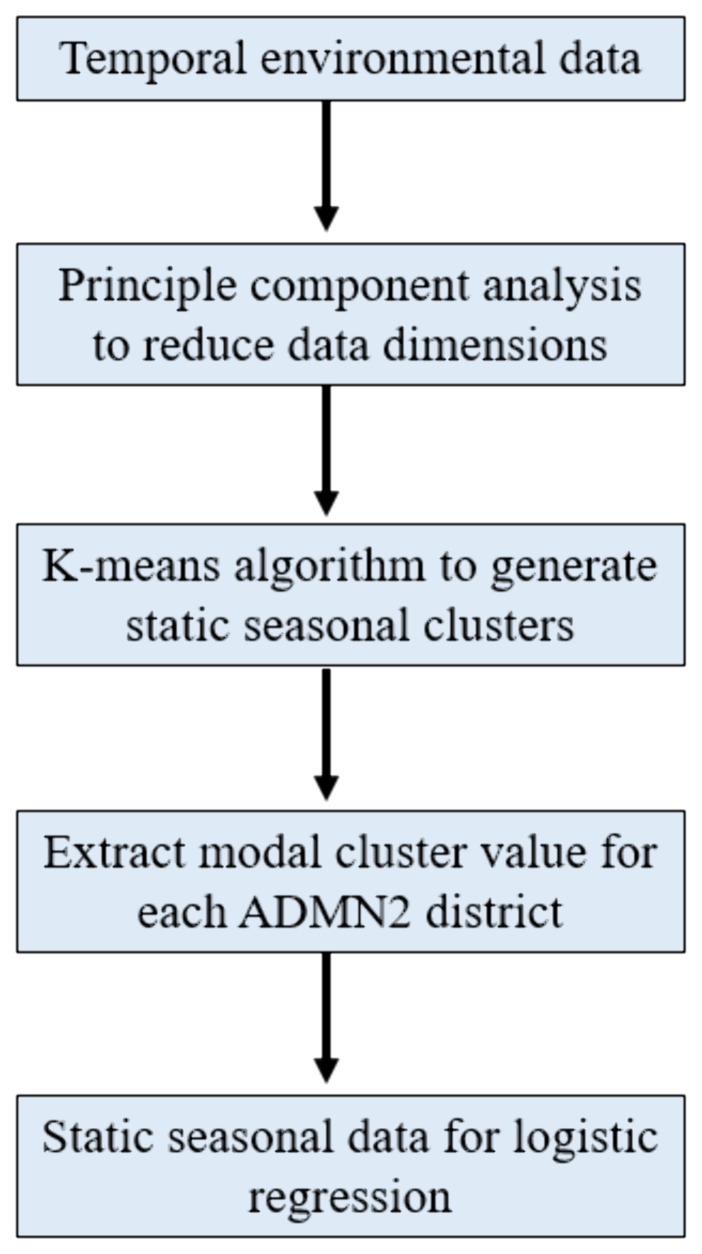
Workflow of environmental data processing for inclusion within static logistic regression model.

For consistency with the methodology used in Molesworth *et al.*’s analysis, we followed the same approach in processing climatic data. This involved processing temporal environmental data into static variables that capture seasonal patterns, which could then be included in a logistic regression model. Monthly means of each temporal environmental variable were converted into a single composite surface representing seasonal profiles. This transformation was applied to rainfall, wind speed, specific humidity, temperature and aerosol optical depth (AOD) data as these variables have a known seasonal component and are expected to change over time. We presumed that land cover category would remain static over the 2003–2022 study period. The workflow, mirrors that implemented by the ADDAPIX software within Molesworth *et al.*’s 2003 analysis ( Griguolo & Santacroce, 1996) ( Figure 1).

**Figure 2.**
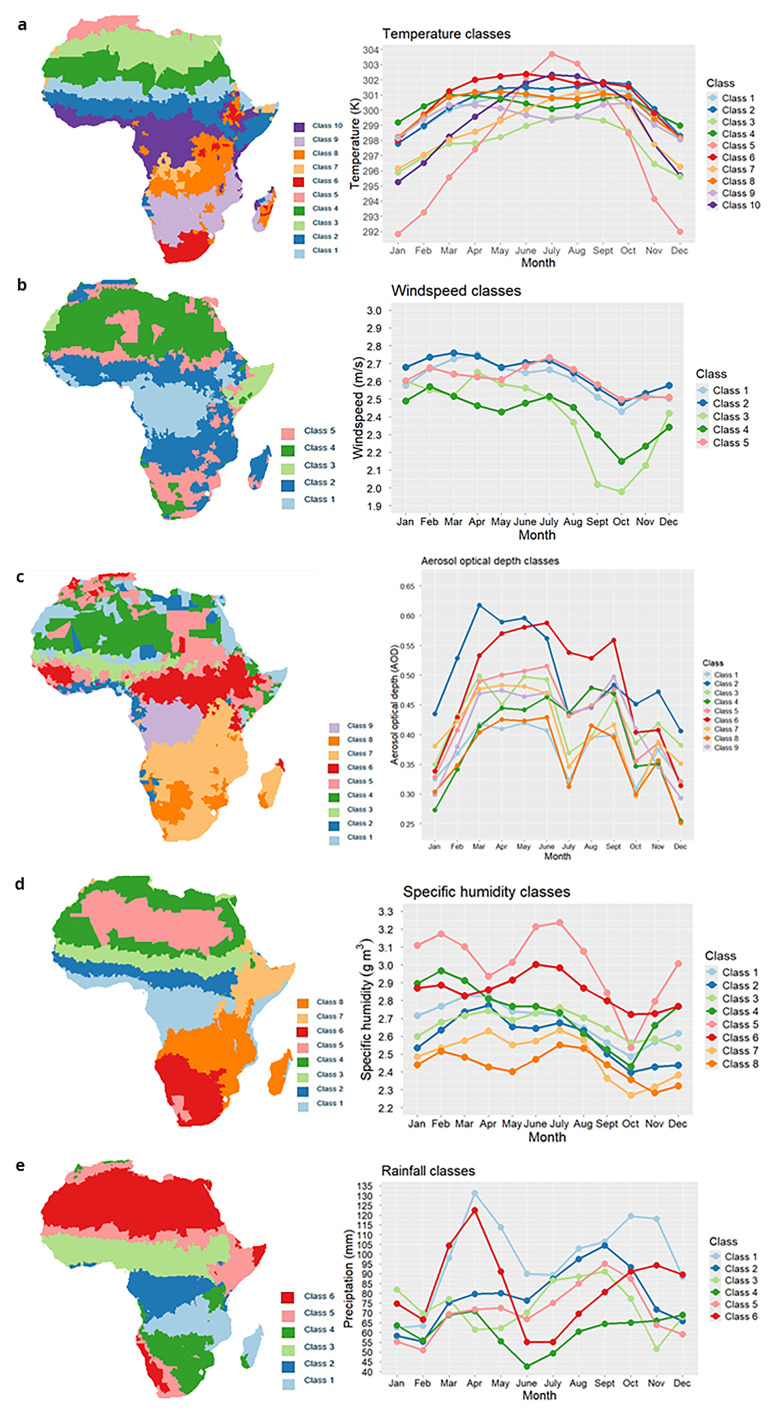
Seasonal cluster profiles of climate (right panel) alongside the relative assigned district classes (left panel). Variables presented were used within the final model: temperature ( **a**), windspeed ( **b**), aerosol optical depth (AOD) ( **c**), specific humidity ( **d**) and rainfall ( **e**).

As an initial step we decreased the data dimensionality of each temporal variable due to the size of the raster data set which would necessitate a high level of computing power. This was achieved using principle component analysis in R 4.3.1 to decrease both the data dimensions and the number of raster layers to be analysed, whilst retaining 95% of the seasonal information retained within the raster data. Following this the K means algorithm was then used to divide each seasonal variable raster into distinct clusters based on data similarity. This is achieved through the K-means algorithm’s ability to assign data points to clusters whilst continuously updating the clusters’ respective mean values, allowing the algorithm to minimize the intracluster variance. For all temporal climate variables (windspeed, humidity, rainfall, temperature and dust) the optimal number of clusters to use in within the k-means clustering algorithm was selected based on the elbow method, plotting total within-cluster sum of squares (SSE) for k = 5–15. We also validated the subjectivity of the elbow method by using silhouette scores for each variable. For all temporal variables, both the elbow curve and silhouette score results can be found in the extended data ( Govender & Sivakumar, 2020; Griguolo & Santacroce, 1996; Umargono *et al.*, 2020). The extended data contains information of the fixed random seeds for all variables used, as well as elbow curves for reproducibility. K-means clustering was initialised using the k-means package algorithm with 25 random starts (nstart = 25) to ensure stability. The algorithm iterated until convergence with a maximum of 300 iterations. Cluster results were validated through an initial visual check against Koppen Geiger and Schultz climate zonal maps alongside Molesworth *et al.*’s environmental clustering results ( Beck *et al.*, 2018). Figure 2 demonstrates the results of our environmental clustering profiles for temperature, windspeed, rainfall, AOD and specific humidity.

Finally, for each of the 6350 ADMN2 districts within the GADM shapefile of Africa, the extract () function from the raster package in R, identified the most common cluster value within each district for all processed environmental variables ( Hijmans *et al.*, 2025). This value was then assigned to each ADMN2 district as its representative cluster. For the landcover data, the extract function was used to determine the most common landcover type within each ADMN2 district in the GADM shapefile. This processing was carried out at the native resolution of each environmental dataset, except for when raster aggregation had occurred, as specified in the supporting information.

For all variables any environmental clusters with less than 50 districts were assigned to their nearest neighbour cluster using a cluster dendrogram to prevent complete separation and inflated significance. This led to a total of 5 windspeed clusters, 6 rainfall clusters, 8 specific humidity clusters, 9 AOD clusters and 10 temperature clusters across the African continent. These k-mean cluster profiles of each variable could then be used as dependent variables within our logistic regression model. This allows for consistency with Molesworth *et al.*’s analysis, with logistic regression allowing for direct comparison between risk maps. By using logistic regression we can examine the impact of environmental risk factors across the whole of the meningitis belt and its surrounding area.

### Model specification and epidemic weighting

**Figure 3.**
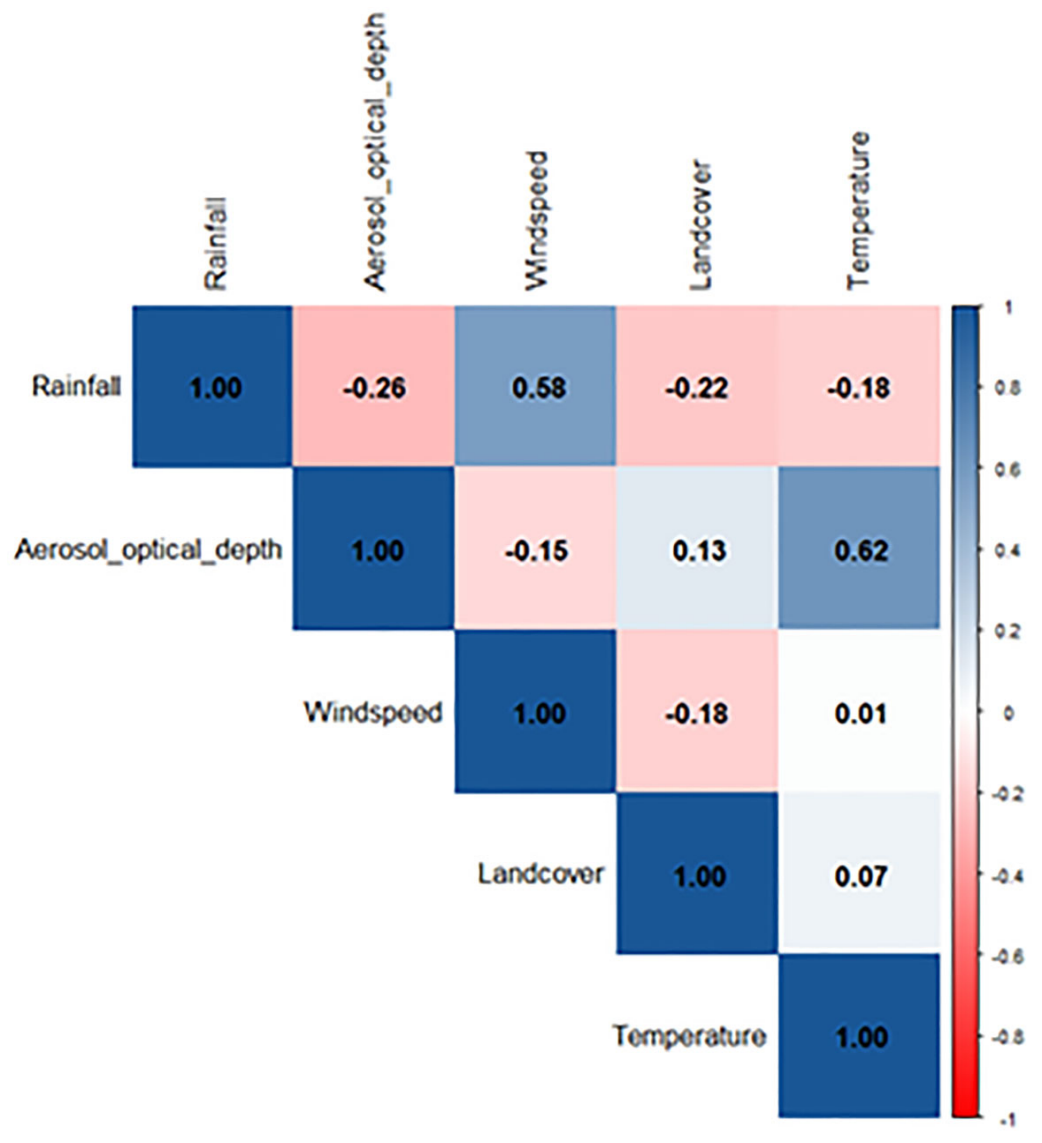
Pearson’s correlation coefficient between climatic variables used within logistic regression (−1 to 1).

Within our study, the dependent variable was district history of meningitis epidemic, whilst the independent variables were k-means clusters of rainfall, aerosol optical depth, specific humidity and wind speed, and average land cover class. Using a cut off value of >0.6 in our Pearson’s correlation coefficient we excluded temperature from our logistic regression model and retained atmospheric dust . With the removal of temperature from the initial model, the variance inflation factor for all variables was under 2.0. ( Figure 3).

**We ran the model weighting for inverse district size**, population density and growth and fertility rate. We balanced the class weights for districts with/without epidemics by ensuring that resulting model sensitivity and specificity are both maximized. This was carried out due to the large imbalance between ADMN2 districts with/without an epidemic. Area averages were also based on a cosine of latitude weighting due to our use of regular longitude/latitude gridded data. We also conducted a sensitivity analysis, running the logistic regression model without the Democratic Republic of Congo (DRC), due to the country’s very limited laboratory confirmation of suspected cases ( Mazamay *et al.*, 2020).

### Risk analysis

Backwards stepwise logistical regression was used to identify the risk of meningitis outbreak within a district in Africa and the relative environmental associations across the whole dataset. For environmental variables, we used a threshold significance level of <0.05 when identifying statistically significant associations. The probability of each district ever having had an epidemic was predicted through the model, before being grouped into risk categories and then mapped. The model was tested by checking specificity and sensitivity, examining collinearity through the Variance inflation factor, as well as utilising McFadden's Pseudo-R2 Interpretation for goodness of fit ( Hardin & Hilbe, 2007). Finally, a receiver-operator characteristics (ROC) curve was used to examine the model's capability to discriminate between true positives and false positives across all possible thresholds. The logistic regression model’s ability to predict outbreaks was verified using mean validation, splitting the data into “train” and “test” sections. Although Molesworth et al, used a 60:40 split for train and test data, we initially selected a 80:20 split as we had a much smaller proportion of ADMN2 districts effected by bacterial meningitis epidemics. We validated our results using a 60:40 split to allow for an equitable comparison with Molesworth’s work. Models were recreated with the train data 10 times and then used to predict epidemic risk within the test data. Mean sensitivity and specificity of the validation data were compared against that of the backward stepwise model formed from the whole data set. R code used to run this analysis can be found within the following GitHub repository ( https://github.com/molly-cliff/Meningitis-belt-location).

## Results

### Epidemic overview and geographic distribution

**Figure 4.**
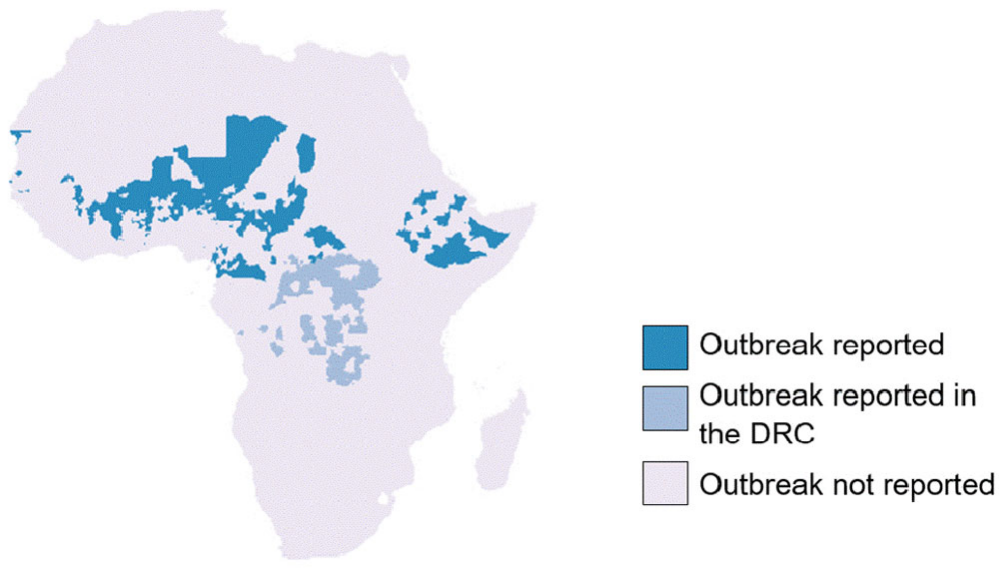
Map of Africa showing the location of meningitis epidemics reported to WHO-AFRO between 2003–2022.

Between 2003 and 2022 there were 1069 reported district level epidemics each with at least 10 suspected cases per 100,000 population per week. Within the same period 14 district-level annual outbreaks were recorded each with a seasonal cumulative incidence of at least 100 suspected meningitis cases per 100,000 population. 55 epidemics were dropped from the final dataset as we were unable to assign them to an appropriate ADMN2 district in either the WHO or GADM shapefile. After considering duplicate districts (with more than one epidemic) 523 districts had experienced at least one outbreak of bacterial meningitis during the study period. This represents 8.2% of all 6350 districts in Africa within our dataset ( Figure 4).

### Regional trends and repeated outbreaks

The majority of bacterial meningitis epidemics within Africa, occurred within the Central Sahel region, with Burkina Faso, Chad and Niger having over 50% of their ADMN2 districts affected by an outbreak. Additionally, within this region, Nigeria had over 200 districts affected by a meningitis outbreak. However, across the most western and eastern parts of the Sahel, Mauritania and Sudan both reported zero outbreaks with Mali also reporting a low number of affected districts. Across the remainder of the meningitis belt Togo, Ghana, The Gambia, and Ethiopia were also affected by meningitis outbreaks. Whilst the north of the Democratic Republic of Congo was thought to be the region of the country within the meningitis belt, between 2003 and 2022 outbreaks occurred throughout the country. Within our study period, 51% of all districts within the DRC experienced a meningitis outbreak. If districts in DRC are excluded then there were 442 ever-affected epidemic districts in our dataset.

**Figure 5.**
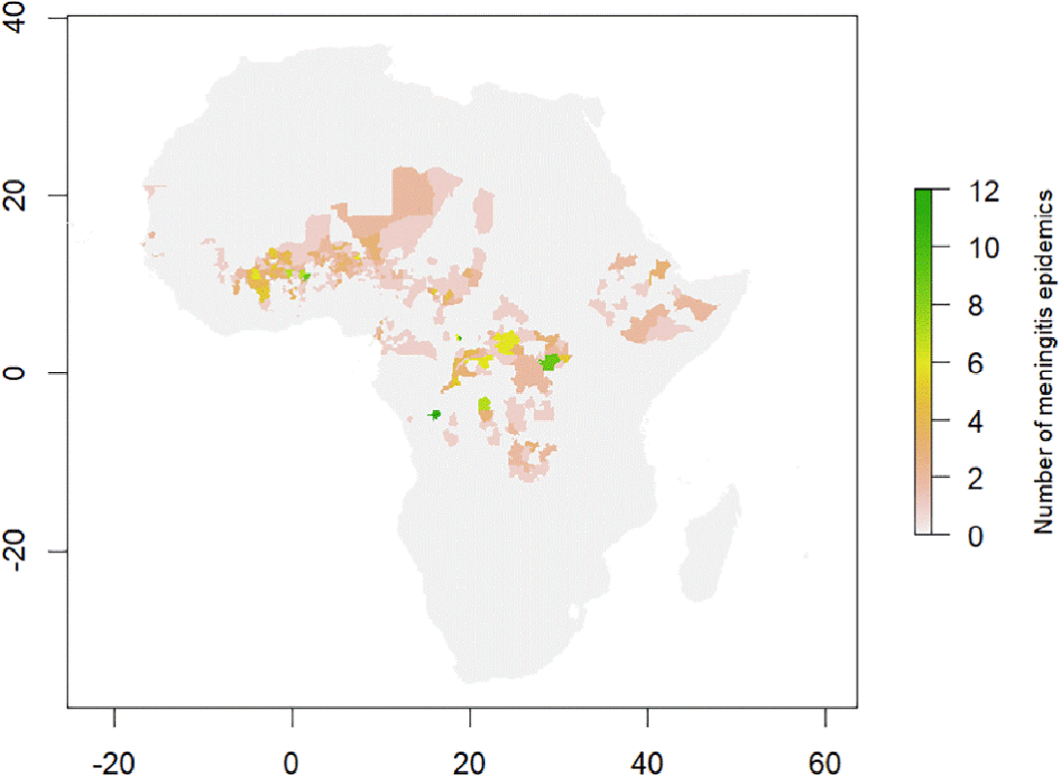
Map of number of bacterial meningitis epidemics per ADMN2 districts across reporting countries (2003–2022).

Figure 5 presents a temporal analysis of districts within Africa that have repeatedly reported epidemics of bacterial meningitis. Whilst most districts within the meningitis belt are reporting less than 5 outbreaks within the 19 year reporting period, a small number of districts are reporting regularly repeating outbreaks. At least one ADMN2 district within each of Niger, the DRC, Central African Republic, Chad, Burkina Faso, Côte d'Ivoire and Benin have reported at least 5 outbreaks from 2003–2022. Table 2 contains climatic class information for the 20 districts with the largest attack rate based on suspected cases counts.

**Table 2.** Climatic classes of each of the ADMN2 districts reporting the 20 largest outbreak weeks of bacterial meningitis.

Country	ADMN2 District	Suspected cases per 100,000 population	Rainfall class	Humidity class	Aerosol optical depth class	Windspeed class
Democratic Republic of the Congo	Manono	1063.56	Class 1	Class 8	Class 7	Class 1
Democratic Republic of the Congo	Mweka	408.97	Class 2	Class 1	Class 10	Class 1
Democratic Republic of the Congo	Banalia	404.08	Class 2	Class 1	Class 6	Class 1
Cameroon	Moutourwa	380.99	Class 3	Class 3	Class 5	Class 2
Democratic Republic of the Congo	Djugu	291.67	Class 2	Class 1	Class 7	Class 1
Nigeria	Aleiro	238.38	Class 3	Class 3	Class 3	Class 2
Burkina Faso	Bazèga	237.26	Class 3	Class 3	Class 3	Class 2
Nigeria	Buji	229.05	Class 3	Class 3	Class 3	Class 2
Niger	Niamey	218.91	Class 5	Class 3	Class 3	Class 5
Nigeria	Jama'are	216.39	Class 3	Class 3	Class 3	Class 2
Nigeria	Taura	190.17	Class 3	Class 3	Class 1	Class 2
Burkina Faso	Kompienga	174.75	Class 3	Class 2	Class 3	Class 5
Burkina Faso	Loroum	173.09	Class 5	Class 3	Class 3	Class 5
Burkina Faso	Kourwéogo	167.41	Class 3	Class 3	Class 3	Class 2
Burkina Faso	Koulpélogo	147.60	Class 3	Class 2	Class 3	Class 2
Democratic Republic of the Congo	Kinshasa	142.24	Class 2	Class 1	Class 10	Class 1
Democratic Republic of the Congo	Mambasa	136.27	Class 2	Class 1	Class 6	Class 1
Cameroon	Ngoumou	132.06	Class 2	Class 1	Class 2	Class 1
Nigeria	Danja	129.59	Class 3	Class 3	Class 3	Class 2
Burkina Faso	Boulkiemdé	128.79	Class 3	Class 3	Class 3	Class 2

In the post-vaccination era, the Democratic Republic of Congo (DRC) has reported over 10 districts within meningitis outbreaks each year from 2011 onwards, highlighting a change in reporting practice. This is notable as it surpasses the typical reporting regions such as the Sahelian countries like Burkina Faso, Chad, and Nigeria, known for many large outbreaks. Burkina Faso, DRC and Nigeria all reported over 100 outbreaks of bacterial meningitis from 2003–2022 with DRC and Nigeria reporting 228 and 316 outbreaks respectively.

### Environmental predictors of meningitis epidemics

All climatic variables were independently associated with the location of epidemics. We initially carried out backwards stepwise regression to decide whether any climatic variables could be removed from the final model. The step function which we used to carry backwards stepwise regression decides the removal of variables based on changes in the Watanabe-Akaike Information Criterion, aiming to create a more parsimonious model. Of all environmental variables considered, removal of landcover was the only change that led to decrease in WAIC, leading to its removal from the final model. We then examined collinearity between variables, using the variance inflation factor. All variance inflation factor (VIF) values were below 2.0, indicative of limited collinearity between variables. Finally, we used the likelihood ratio test to evaluate the contribution of each remaining predictor, comparing nested models with and without remaining predictors. A statistically significant change in model fit was defined as p < 0.05. Alongside this we compared the area under the receiver operating characteristic curve (AUC) between nested models to see which model predictors lead to a notable AUC decrease (>0.05) upon their removal.

Comparative details of model specificity and sensitivity, including the landcover cluster variable can be found within the extended data. Despite the sensitivity and specificity of the model, including landcover remaining relatively close to that of the final model, eliminating the variable allowed for a more parsimonious model.

Following this methodology, rainfall, aerosol optical depth, windspeed and humidity were used as independent variables within the final model. Weighting for district size, population density, and fertility rate did not improve the model performance and was therefore discarded. In manually balancing the class weighting for the response variable through ensuring that the resulting model sensitivity and specificity are both optimised, a weighting of 0.842 for districts with an epidemic against a weighting of 0.158 for districts without an epidemic was most appropriate.

### Model performance

 Our final backwards stepwise model including the DRC, is outlined in Table 3 in relation to its estimated coefficients, 95% confidence intervals, and variable contribution. Reference clusters were chosen in order to balance standard error across the logistic regression model. We aimed to avoid the risk of inflated standard errors which would undermine model precision, instead choosing clusters based on the dependent variable distribution.

**Table 3.** Logistic regression results regarding the association between meningitis outbreaks and climatic clusters. Variables selected in backwards stepwise regression for final model were rainfall, aerosol optical depth, windspeed and specific humidity.

Variable (Class)	Experienced an epidemic (n)	Did not experience an epidemic	Odds ratio	95% CI Lower	95% CI Upper	p-value
Rainfall - Class 1	12	545	3.779	0.998	14.311	0.0504
Rainfall- Class 2 (Reference)	58	813	1	Reference	Reference	Reference
Rainfall - Class 3	369	1312	1.196	0.604	2.368	0.607
Rainfall -Class 4	0	1057	0.006	0.0004	0.0004	0.104
Rainfall - Class 5	77	1400	0.392	0.168	0.915	0.0303
Rainfall - Class 6	7	700	0.047	0.012	0.187	<0.0001
Aerosol -Class 1	60	505	3.895	1.612	9.411	0.0025
Aerosol - Class 2 (Reference)	15	600	1	Reference	Reference	Reference
Aerosol - Class 3	231	242	6.258	2.631	14.886	<0.0001
Aerosol - Class 4	6	307	2.9	0.767	10.961	0.1166
Aerosol - Class 5	75	1179	1.645	0.714	3.787	0.2421
Aerosol - Class 6	70	1204	1.913	0.856	4.277	0.1141
Aerosol - Class 7	20	1406	0.294	0.095	0.908	0.0335
Aerosol - Class 8	0	103	0.783	0.028	22.175	0.8858
Aerosol - Class 9	46	281	4.902	2.092	11.485	0.0003
Humidity - Class 1	88	1268	0.075	0.037	0.153	<0.0001
Humidity - Class 2	114	424	0.369	0.216	0.631	0.0003
Humidity - Class 3 (Reference)	273	930	1	Reference	Reference	Reference
Humidity - Class 4	6	1382	0.062	0.023	0.166	<0.0001
Humidity - Class 5	3	92	2.616	0.439	15.584	0.291
Humidity - Class 6	0	242	0.248	0.01	5.995	0.3906
Humidity - Class 7	23	562	0.193	0.086	0.432	<0.0001
Humidity - Class 8	16	927	0.076	0.021	0.274	0.0001
Wind Speed - Class 1 (Reference)	103	950	1	Reference	Reference	Reference
Wind Speed - Class 2	309	3122	0.359	0.212	0.61	0.0001
Wind Speed - Class 3	2	109	0.381	0.073	1.979	0.2509
Wind Speed - Class 4	12	590	0.156	0.055	0.448	0.0005
Wind Speed - Class 5	96	1056	0.347	0.155	0.776	0.01

### Epidemic risk mapping across Africa

**Figure 6.**
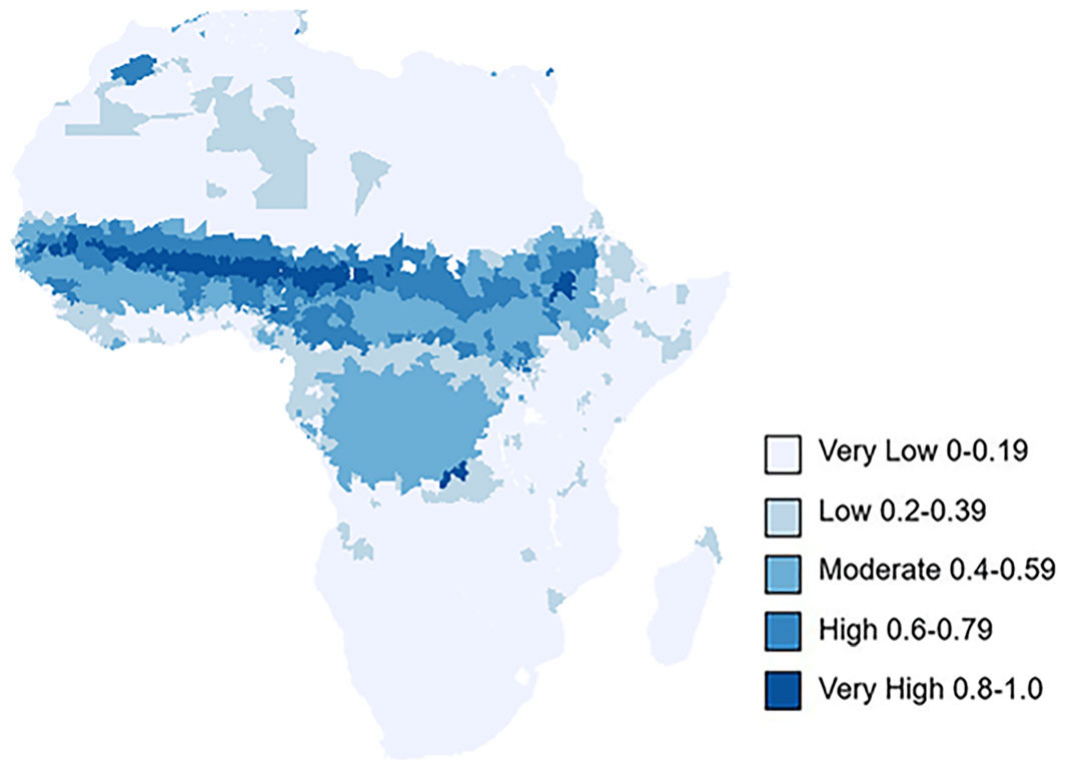
Risk map of meningitis outbreaks across Africa, based on our final logistic regression results. The key represents the probability of ADMN2 district having experienced a meningitis outbreak based on environmental cluster data from 2003–2022. The extended information contains 95% CI risk maps of the probability of ADMN2 district having experienced a meningitis outbreak.

In reference to our risk map and table ( Figure 6, Table 4), the central and western part of the Sahelian region of Africa, with lower rainfall, specific humidity and an extended dry season had the highest risk of meningitis outbreaks (probability >0.8), mirroring Molesworth *et al.*’s observations. Burkina Faso, Cameroon, Chad, Gambia, Mali, Niger, Nigeria, Senegal, South Sudan and Sudan all had at least 25% of their districts with a high risk (probability >0.6), of meningitis outbreaks. This region remained at the highest risk for outbreaks even at the lower bound of the 95% confidence interval. Countries to the south of the Sahel, with a shorter and less extreme dry season had a moderate risk of meningitis outbreaks (probability 0.4–0.59). In contrast to Molesworth *et al.*, several countries to the south were identified to have a moderate risk of meningitis outbreaks. Republic of the Congo, Gabon, and Angola all had at least 15% of their districts with a moderate risk of meningitis outbreaks. Notably Gabon and the Republic of Congo had 68.75% and 29.73% districts with a moderate risk of outbreak. In addition to this Algeria had 108 of its southernmost districts with a moderate risk of outbreaks. These are all countries border the current meningitis belt. Whilst this may suggest an expansion of the meningitis belt more work should be undertaken to verify the reported meningitis outbreaks, specifically with respect to laboratory confirmation of *Neisseria meningitidis.*

**Table 4.** Number of districts within each risk category for all countries across Africa. The key represents the probability of ADMN2 district having experienced a meningitis outbreak based environmental cluster data from 2003–2022.

Country	Very Low 0.0–0.19	Low 0.2–0.39	Moderate 0.4–0.59	High 0.6–0.79	Very High 0.8–1.0
Algeria	1324	20	108	0	0
Angola	121	16	26	0	0
Benin	46	3	22	5	2
Botswana	30	0	0	0	0
Burkina Faso	0	0	11	12	22
Burundi	130	0	0	0	0
Cameroon	2	24	9	19	4
Central African Republic	0	17	24	10	0
Chad	9	1	17	24	2
Côte d'Ivoire	16	7	10	0	0
Democratic Republic of the Congo	42	50	144	2	2
Djibouti	17	3	0	0	0
Egypt	337	0	1	2	0
Equatorial Guinea	29	0	3	0	0
Eritrea	34	12	3	1	0
Ethiopia	18	20	33	4	3
Gabon	7	18	11	1	0
Gambia	0	9	8	20	0
Ghana	180	20	30	30	0
Guinea	7	4	17	66	3
Guinea-Bissau	3	0	34	0	0
Kenya	261	37	0	0	0
Lesotho	10	0	0	0	0
Liberia	10	52	3	0	0
Libya	22	0	0	0	0
Madagascar	20	2	0	0	0
Malawi	243	0	0	0	0
Mali	8	1	10	20	11
Mauritania	18	6	13	7	0
Morocco	42	9	0	3	0
Mozambique	122	6	0	0	0
Namibia	107	0	0	0	0
Niger	6	1	1	24	4
Nigeria	159	101	154	162	193
Republic of the Congo	3	111	33	1	0
Rwanda	30	0	0	0	0
Senegal	0	13	19	11	2
Sierra Leone	4	9	0	1	0
Somalia	65	9	0	0	0
South Africa	52	0	0	0	0
South Sudan	1	1	27	16	0
Sudan	32	4	24	19	0
Tanzania	168	8	0	0	0
Togo	18	3	7	12	0
Tunisia	114	121	1	1	0
Uganda	127	23	7	6	0
Western Sahara	4	0	0	0	0
Zambia	115	0	0	0	0
Zimbabwe	91	0	0	0	0
**Total**	**4203**	**672**	**810**	**420**	**245**

### Logistic regression model sensitivity and specificity

**Figure 7.**
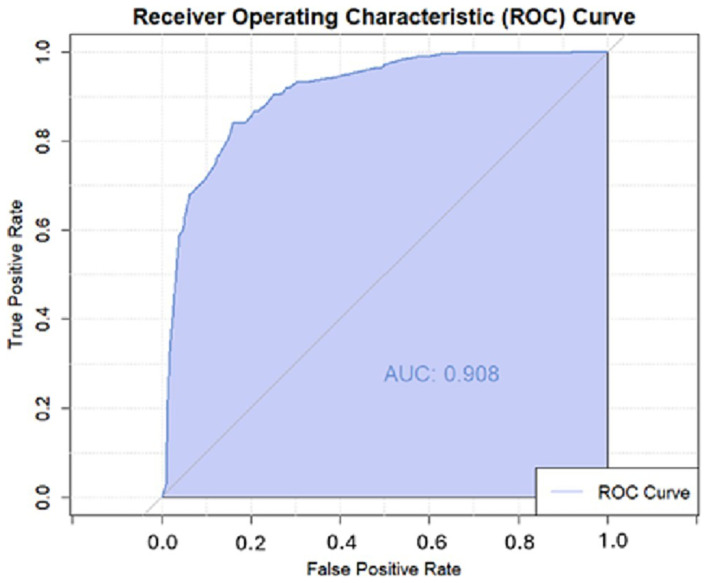
Receiver Operating Characteristic Curve for climatic logistic regression.

We used a receiver operator curve to understand our model’s performance regarding sensitivity and specificity independent of a single probability cutoff ( Figure 7). The area under the curve represents the model's ability to distinguish between true positives and false positives across all possible classification thresholds. With an area under the curve (AUC) of 0.908, the epidemic risk assigned was higher for districts with epidemics than for those without in 90.8% of ADMN2 districts. In using a probability cutoff value of >0.4 for predicting epidemic experience, the model had a specificity and sensitivity of 82.07% and 82.22%, respectively; these statistics were confirmed in the validation process ( Table 5). Here, specificity and sensitivity refer to the model’s ability to correctly identify ADMN2 districts that have never/ever been affected by a meningitis epidemic, as in true negatives and true positives. As a comparison, Molesworth *et al.*, achieved a relative specificity and sensitivity of 67% and 83% within their full model using a cut-off value of >0.4. When the intersection overlap thresholds were adjusted to 60% and 40% as opposed to the previously used 50% and 30%, the AUC remained at 0.908, as only four district outbreaks were affected by the small boundary changes.

**Table 5.** Results of sensitivity and specificity of final model for full dataset and mean validation. Final model included cluster profiles of rainfall, aerosol optical depth, and specific humidity, with the mean validation using a 80%/20% and 60%/40% train and test split.

Epidemic experience	Model Predicted		
Never	4782	1045	5827
Ever	93	430	523
Total districts	4875	1475	6350
**Model**	**Specificity % (95% CI)**		**Sensitivity % (95% CI)**
Final (100%)	82.07		82.22%
Mean validation (20%)	81.66(80.13-84.27)		82.21(80.141-84.28)
Mean validation (40%)	82.03(81.71-82.35)		82.38 (80.64-84.11)

### Sensitivity analysis: Exclusion of DRC

The results of the logistic regression model without DRC can be found in the extended data. These results include a full model table, risk category table (Extended data), ROC curve (Extended data) and sensitivity and specificity table. Overall, removing the DRC improved model fit with a sensitivity of 87.33% and specificity of 87.66 %, when using a probability cutoff value of >0.4 for predicting epidemic experience. This represents a 5% increase in both model sensitivity and specificity. This can also be seen in the ROC curve with an improved AUC of 0.932.

Within this model, the proportion of districts with a high probability of meningitis outbreaks remained largely comparable to our full model. The proportion of ADMN2 districts with a high risk of meningitis (probability >0.66) was 6.6% which is comparable to 6.0% ADMN of districts in this risk category in our full model. Burkina Faso, Cameroon, Chad, Gambia, Mali, Niger, Nigeria, Senegal, South Sudan and Sudan all had at least 25% of their districts with a high risk of meningitis outbreaks. Notably, Burkina Faso and Niger had 76% and 78% respectively of their ADMN2 districts within this risk category and Nigeria had 355 districts classified as at least high risk.

 The key difference within the sensitivity analysis is that a large number of ADMN districts that would have been categorized as moderate risk (probability 0.4–0.59) within the full model are now low risk (probability 0.2–0.39). Within this model, no country on the periphery of the meningitis belt had any districts with at least a moderate risk of meningitis outbreak (probability 0.4–0.59). Republic of the Congo, Gabon, and Angola which were all at risk of meningitis outbreaks within the full model, had no districts with a moderate risk of meningitis outbreaks.

### Sensitivity analysis: Exclusion of post-MenAfriVac outbreaks

When removing outbreaks that occurred post the introduction of MenAfriVac across Africa, we were left with 308 districts ever affected by a meningitis outbreak. This led to reduced model sensitivity (74.2%), compounded by higher specificity (93.6%), with an AUC of 0.929 using a 0.4 ROC cut-off. Further details of this analysis, alongside a risk map of the subsetted outbreak model can be found within the extended data. When only considering pre-MenAfriVac epidemics the region at risk for meningitis outbreaks within our risk map had contracted. Regions of countries previously identified as being at risk of meningitis epidemics, including parts of Ethiopia, Eritrea, Ghana, Togo and the Democratic Republic of the Congo, no longer had a demonstrable outbreak risk.

## Discussion

### Key findings

This study examines the spatial patterns of meningitis epidemics across the African continent from 2003–2022 in relation to environmental variables. We determined that rainfall, specific humidity, windspeed, and aerosol optical depth were key predictors in identifying outbreak location. Our model surpasses Molesworth *et al.*’s in environmental raster resolution and has equal if not improved statistical specificity and sensitivity. Our environmental logistic regression model has significantly improved specificity, at 82.1% as opposed to Molesworth *et al.*’s 67.0%. Through maintaining methodological comparison with Molesworth et al’s original work we have conducted a longitudinal epidemiological evaluation of climatic effects on meningitis incidence without introducing confounding factors through methodological changes. Our study extends the temporal window of Molesworth’s previous analysis by an additional 20 years. As a comparison Savory’s analysis only extended the analysis by only four years, examining 71 meningitis epidemics whereas our analysis includes 523 outbreaks, representing a substantially richer dataset. Additionally, we considered the impact of wind speed on bacterial meningitis epidemics a variable not examined in either Molesworth’s or Savory’s work. Finally, with 20 years having passed since the Molesworth and Savory’s publications we have been able to take advantage of the considerable increase in spatial and temporal resolution of environmental data.

Our study reaffirmed the key influence of climate on meningitis outbreak risk, across the belt. As in Molesworth *et al.*’s work, districts/regions of Africa with distinct wet and dry seasons were more likely to have meningitis outbreaks. This is highlighted within our specific humidity clustering, where Clusters 2 and 3 which had consistently low humidity from January to March, within the meningitis dry season had the most outbreaks. While there is some variation in aerosol optical depth preceding an outbreak, Cluster 2 which had the highest wind speed within the typical meningitis dry season had the majority of outbreaks. All environmental variables exhibited seasonal clustering within or around the meningitis belt, emphasizing the climatic influence on meningitis epidemics. Importantly, our full model highlighted a potential expansion of the meningitis belt as a result of a changing climate. Republic of the Congo, Gabon and Angola all had at least 15% of their districts with a moderate risk (probability 0.4–0.59) of meningitis outbreaks. Within our study period, Angola experienced several meningitis outbreaks, indicative of a potential expansion of the region at risk ( Savory *et al.*, 2006). More recently, in 2023 Angola experienced a significant increase in suspected meningitis incidence, reporting over 68,000 cases. This is also supported by Savory *et al.*, who when evaluating Molesworth’s environmental data with a 100-mile southwards expansion of risk region were able to improve the pre-existing environmental model sensitivity from 84% to 88% ( Savory *et al.*, 2006). However, our sensitivity analysis, excluding data from the Democratic Republic of the Congo (DRC), demonstrated the model’s vulnerability to inconsistent case reporting. Within the DRC very few suspected cases are tested for laboratory confirmation ( Mazamay *et al.*, 2020). Across rural ADMN2 districts in Africa, suspected bacterial meningitis are infrequently confirmed as a result of limited access to laboratory and hospital diagnostic facilities ( Mazamay *et al.*, 2020).

Published literature, describing both the structure and performance of the meningitis surveillance system utilized across the DRC, remains limited. This ultimately makes it difficult to assess surveillance system discrepancies, reporting biases and spatial misclassification. However, a 2021 bacterial meningitis outbreak within the country highlights several systemic weaknesses within routine surveillance, likely to influence spatial risk estimates ( Bita Fouda *et al*., 2024). Within this outbreak, only 213 cerebrospinal fluid specimens were laboratory tested from over 2500 reported cases. Of the samples tested, 57 (26.7%) were confirmed as bacterial meningitis. To contrast, approximately 800 cases were tested with a rapid malaria diagnostic test, of which 77 tested positive, leading to a 5-week delay in epidemic detection. During this period, samples were tested for Ebola virus, shigellosis and salmonellosis at the National Institute for Biomedical Research (INRB) laboratory in Kinshasa ( Bita Fouda *et al*., 2024). Such ongoing testing for other pathogens during the meningitis epidemic suggests that limitations in meningitis testing were likely due to a lack of reagents, experience, or appropriate training. Across the DRC this warrants an investigation into reporting practices as well as pathogen-wide laboratory testing.

When we excluded the DRC from our logistic regression model, there was a notable increase in sensitivity and specificity by approximately 5%. However, within this model, no countries adjacent to the existing meningitis belt had a demonstrated risk of outbreaks, highlighting the importance of data quality and its impact on perceived risk patterns. In line with the Defeating Meningitis by 2030 Roadmap, developing reliable and affordable diagnostic tests for bacterial meningitis, could improve case ascertainment within resource-limited settings ( Cliff *et al.*, 2024). Improved surveillance of meningitis outbreaks across Africa is vital to validate the potential expansion of high-risk areas and deepen our comprehension.

### Limitations and future research

There are some limitations regarding our modelling approach, the most pertinent being our choice to only use environmental predictors. These provide necessary but not sufficient conditions for an outbreak. A number of risk factors including vaccination rates, population level immunity, migration levels and physical contact rate have a key role within the dynamics of bacterial meningitis outbreaks across Africa ( Agier *et al.*, 2017; Trotter *et al.*, 2017). Increasing vaccination rates and population immunity would likely decrease meningitis risk. Similarly, lower regional levels of physical contact between individuals would also decrease bacterial meningitis risk, with lower risk also being found in less urbanized areas. Higher levels of migration may also compound the increasing risk of bacterial meningitis outbreaks. Whilst not being able to include these variables within a static logistic regression model may overstate the importance of environmental factors, it is unlikely that these socioeconomic variables would be available on a continental level at an appropriate resolution for inclusion within the model. Furthermore, data regarding either meningococcal or pneumococcal vaccination rates would likely be coarse and heterogenous within its coverage.

Additionally, while used static land cover as a risk factor within our model, further work could consider this as a temporal variable due to the impact of deforestation and desertification on outbreak risk ( Tollefson, 2020; Yang *et al.*, 2022). As in Molesworth *et al.*’s analysis, whilst we received epidemic reports from enhanced disease surveillance countries, other countries outside this region may have unreported meningitis outbreaks. Through combining local data into ADMN2/ADMN1 statistics there is the potential for some discrepancy between regions where patients develop bacterial meningitis and the location of health facilities reporting cases, leading to a loss of data specificity ( Molesworth *et al.*, 2003). The use of k-means clustering and PCA simplifies the data being used, potentially compromising the predictive power of the logistic regression model, if key variations in the data are not captured. We avoided particularly sparse categories by ensuring at least 50 districts were in each cluster but some categories inevitably described more districts than others. In the future we will explore different methods that do not involve k-means clustering, but found it useful here to repeat the methods used previously to explore any signals for changing geographic risk.

Within our analysis we excluded vaccination coverage as a risk factor within our primary model, as it will not have an impact on environmental risk factors for meningitis across Africa. Although vaccination will serve to decrease the risk of epidemics in vaccinated areas it will not affect unvaccinated regions and is independent of environmental/climatic variables. MenAfriVac, offering protection against group A meningococcus, was introduced in mass campaigns across the belt from 2010 onwards, followed by introduction into the essential programme of immunisation (EPI) ( Djingarey *et al*., 2015). Most countries have also introduced pneumococcal conjugate vaccines into EPI over a similar time period. To account for how the introduction of both pneumococcal and meningococcal vaccines during our study period has reshaped the epidemiology of bacterial meningitis, we conducted a sensitivity analysis only considering outbreaks that had occurred before the introduction of MenAfriVac. Details of this can be found within the extended data, with the resulting risk map showing a decrease in the area at risk of meningitis epidemics. This suggests that several outbreaks that occurred across the belt were likely caused by non-serogroup A meningococci or other meningitis-causing bacteria.

Lastly, although we used a 20-year time period as such length is typical in climate analyses, we assumed that climatic conditions were static across the reporting period. This may not be the case. Nicholson *et al.*, have determined that there has been a decrease in precipitation levels preceding the typical dry season across the Sahel ( Nicholson *et al.*, 2018). Within this region temperatures are also rising 1.5 times faster than the global average ( Office of the High Commissioner for Human Rights, 2025). Ghanaian focus group discussion has highlighted participants' experience of an expanding dry season which now stretches from February to June, with air temperatures rising to 48°C ( Codjoe & Nabie, 2014). It has also been suggested that there has been a global increase in the severity and intensity of sand and dust storms including the Harmattan winds over Africa in recent decades ( Li *et al.*, 2025), contributing to Sahelian drought persistence and increasing the risk of meningitis outbreaks. These findings reinforce the need for more sophisticated spatial-temporal modelling to anticipate how climate change may reshape meningitis epidemiology.

## Conclusion

Our study highlights that the environmental risk factors for meningitis epidemics have largely remained unchanged across a 40-year period, aligning with previous findings by Molesworth *et al.* Whilst our logistic regression model suggests a potential expansion of the meningitis belt, this signal is highly sensitive to the quality of surveillance data. The inclusion of the DRC, where we know that few cases are laboratory confirmed had a significant impact on the model. Whilst Republic of the Congo, Gabon, and Angola had a moderate risk of meningitis (probability 0.4–0.59) within our full model, no countries surrounding the meningitis belt had a demonstrated risk of meningitis within the sensitivity analysis that excluded DRC. It is essential to improve epidemic reporting and diagnostic capacity within meningitis outbreaks, to accurately assess disease risk across the continent.

## Data Availability

Figshare: Updating the meningitis belt: associations between environmental factors and epidemic meningitis risk across Africa- Supporting data https://doi.org/10.6084/m9.figshare.29900975 ( Cliff, 2025a). This project contains the following underlying data:
Figure2_rainfall.xlsx, Figure2_dust.xlsx, Figure2_temperature.xlsx, Figure2_windspeed.xlsx and Figure2_humidity.xlsx- Files containing the climatic variable averages for k mean clustering technique across months. These files are used in plotting the graphs within Figure 2
ADMN2_outbreak_details.xlsx- File containing the ADMN district locations of meningitis epidemic history in Africa from 2003–2022. This file was used to generate the plot in Figure 4
outbreak_counts.xlsx- File containing the number of outbreaks in ADMN districts across Africa from 2003–2022. This file was used to generate the plot in Figure 5
Cluster_epidemic_analysis.xlsx- File containing the final k means cluster values for each of the ADMN2 districts used within the final logistic regression model. This file was used to generate Table 3, Table 4 and Table 5, as well as the relevant sensitivity analysis within the Extended data
Model_epidemic_prediction.xlsx- File containing results of the logistic regression model, and has both the predicted risk and category used within our final predictive risk map. This file was used to generate Figure 6 and supports the results of our full model's sensitivity and specificity Data are available under the terms of the Creative Commons Attribution 4.0 International license (CC-BY 4.0). The raw data on meningitis epidemics by ADMN district was provided by WHO AFRO via co-authors Andre Bita, Anderson Latt and Clement Lingani. These data are not publicly available in the format used in this publication. To access this data an application would need to be made via Clement Lingani who can be contacted at linganic@who.int. A range of publicly available meningitis surveillance data are available via the Power BI WHO meningitis data dashboard Figshare: Updating the meningitis belt: associations between environmental factors and epidemic meningitis risk across Africa- Extended data and additional information **https://doi.org/10.6084/m9.figshare.29786375.v1** ( Cliff, 2025b) This project contains the following underlying data:
Extended data.pdf- This pdf file contains the results of the sensitivity analysis carried out within this paper, where we ran the logistic regression model without the Democratic Republic of Congo, due to the country’s limited laboratory confirmation. Alongside this the risk map of our sensitivity analysis only examining pre-MenAfriVac vacciantions can be found within this data. This pdf file also contains information regarding weighting strategies, data aggregation and the specific humidity calculation used, which are not required to follow study design and analysis of the results, but are instead supplementary. Data are available under the terms of the Creative Commons Attribution 4.0 International license (CC-BY 4.0).
