# Supplementary material for: New title: Re-evaluating environmental associations with meningitis risk across Africa
Source: Wellcome Open Res. 2026 Feb 13;10:497. Originally published 2025 Sep 9. [Version 2] doi: 10.12688/wellcomeopenres.24771.2 (PMC12982989; doi:10.12688/wellcomeopenres.24771.2)
Supplement: Peer review history

# Reviewer report: Reviewer response for version 2

**DOI:** 10.21956/wellcomeopenres.28522.r147683

**Published:** 2026-05-20

**Authors:** Junjun Chen

**Affiliations:** 1 Johns Hopkins University, Baltimore, USA

The authors have satisfactorily addressed my previous concerns. The methodology is now well-documented. I approve this revised version.

Is the work clearly and accurately presented and does it cite the current literature?

Yes

If applicable, is the statistical analysis and its interpretation appropriate?

Yes

Are all the source data underlying the results available to ensure full reproducibility?

Yes

Is the study design appropriate and is the work technically sound?

Yes

Are the conclusions drawn adequately supported by the results?

Yes

Are sufficient details of methods and analysis provided to allow replication by others?

Yes

Reviewer Expertise:

Biomedical engineering, public health data science, environmental epidemiology, and AI-driven modeling of disease risk.

I confirm that I have read this submission and believe that I have an appropriate level of expertise to confirm that it is of an acceptable scientific standard.

# Reviewer report: Reviewer response for version 2

**DOI:** 10.21956/wellcomeopenres.28522.r147684

**Published:** 2026-03-12

**Authors:** Tiantian Li

**Affiliations:** 1 Chinese Center for Disease Control and Prevention, Beijing, China

**Reviewer Report**

 The revised manuscript has satisfactorily addressed the main concerns raised in the previous review round. The authors have improved the transparency and robustness of the analysis by expanding the methodological description of the clustering procedure (including initialization, random seeds, and convergence criteria), providing additional sensitivity analyses (e.g., pre-MenAfriVac outbreaks and district boundary thresholds), and revising the discussion to better contextualize vaccination coverage and surveillance limitations. The revised title more accurately reflects the scope of the study, and the inclusion of uncertainty information in the extended data improves the interpretability of the risk estimates.

 I have only two minor suggestions that may help further improve clarity.

 First, the manuscript should clarify which variables were ultimately retained in the final regression model. The Discussion notes that rainfall, specific humidity, wind speed, and aerosol optical depth were key predictors, whereas Table 5 appears to include cluster profiles of rainfall, aerosol optical depth, and specific humidity only. Clarifying whether wind speed was included in the final model would improve consistency across sections.

 Second, the manuscript notes that variables with Pearson correlation coefficients >0.6 were excluded to reduce collinearity, leading to the removal of temperature while retaining aerosol optical depth. A brief clarification or supporting reference for the chosen correlation threshold and variable selection approach would further improve transparency.

 Overall, the revisions have strengthened the manuscript and improved its reproducibility and interpretation. I consider the manuscript suitable for indexing.

Is the work clearly and accurately presented and does it cite the current literature?

Partly

If applicable, is the statistical analysis and its interpretation appropriate?

Partly

Are all the source data underlying the results available to ensure full reproducibility?

Partly

Is the study design appropriate and is the work technically sound?

Partly

Are the conclusions drawn adequately supported by the results?

Partly

Are sufficient details of methods and analysis provided to allow replication by others?

Partly

Reviewer Expertise:

Mainly engaged in research on environment and health, with a focus on the impacts of climate change, air pollution, and other environmental factors on population health, including the environmental drivers of infectious disease transmission.

I confirm that I have read this submission and believe that I have an appropriate level of expertise to confirm that it is of an acceptable scientific standard.

# Reviewer report: Reviewer response for version 1

**DOI:** 10.21956/wellcomeopenres.27291.r134964

**Published:** 2025-11-19

**Authors:** Tiantian Li

**Affiliations:** 1 Chinese Center for Disease Control and Prevention, Beijing, China

**Reviewer:**

 This study utilizes WHO-AFRO meningitis surveillance data from 2003–2022, combined with multiple environmental covariates including rainfall, specific humidity, aerosol optical depth (AOD), wind speed, and land-cover type, to evaluate environmental determinants of epidemic meningitis risk across Africa. Using K-means clustering and logistic regression, the authors identify rainfall, humidity, and aerosol concentration as the strongest predictors. When data from the Democratic Republic of the Congo (DRC) are included, the model suggests a potential south-western expansion of high-risk areas; however, after excluding the DRC, the results align closely with Molesworth et al. (2003), showing no spatial expansion. The study represents a substantial methodological advance over previous work in terms of temporal coverage, data resolution, and model reproducibility, providing valuable evidence on climate–meningitis relationships. Nevertheless, several aspects concerning the study’s framing, variable selection, model comparability, and interpretation require further refinement.

**Major comments**

 1. The current title, “Updating the meningitis belt,” implies that the research re-defines the spatial boundaries of the belt. Yet, after excluding the DRC, the distribution of high-risk areas remains largely consistent with Molesworth et al. (2003), without any confirmed expansion. This could mislead readers regarding the novelty of the findings. The authors are encouraged to reconsider the title and reposition the study focus. If no new belt boundary has been empirically defined, alternative titles such as “Re-evaluating environmental associations with meningitis risk across Africa” or “Revalidation of environmental determinants of meningitis outbreaks” would more accurately reflect the study’s scope.

 2. The introduction cites Chen et al. (2023), who demonstrated a significant association between temperature variability and meningitis incidence, yet temperature was not included in the present model. The authors should clarify whether this omission was due to multicollinearity, data availability, or model stability. Given temperature’s central role in dry-season transmission mechanisms, its exclusion requires explicit justification based on statistical or theoretical grounds.

 3. The authors deliberately modeled only environmental variables to avoid immunological confounding. However, since the large-scale introduction of MenAfriVac after 2010 has profoundly altered meningitis epidemiology, omitting vaccination coverage, population immunity, migration, and urbanization may overstate the role of environmental predictors. The discussion should explicitly acknowledge this limitation and speculate how inclusion of immunization or socio-demographic variables might alter the observed associations.

 4. The DRC substantially affects spatial risk estimates, yet the explanation provided—“limited laboratory confirmation”—is insufficient. The authors should explore surveillance system discrepancies, reporting biases, and potential spatial misclassification (case reporting site vs infection origin). The Methods section should also specify how spatial mismatches and uncertainty were addressed (e.g., overlap thresholds, interpolation precision, temporal alignment).

 5. The paper’s main contribution lies in: (i) extending the temporal window by 20 years; (ii) using substantially higher-resolution environmental datasets; (iii) maintaining methodological comparability with Molesworth et al. to enable longitudinal evaluation; and (iv) introducing wind-speed as a new variable. These advances should be systematically emphasized in the Introduction and Discussion, with explicit comparisons to Molesworth (2003), Savory (2006), and Tall (2022) to highlight methodological and policy-relevant innovations.

 6. Wind speed and land-cover were excluded from the final model, yet both are ecologically relevant—especially wind speed, which is closely linked to aerosol dispersion during the dry season. The authors should provide detailed information on stepwise-selection criteria (e.g., significance thresholds), multicollinearity diagnostics (e.g., VIF values), and decision rules for exclusion. Supplementary material should include model performance comparisons with and without these variables to improve transparency and reproducibility.

 7.The Methods section notes the use of iterative modeling and the elbow method to determine the optimal number of clusters, but the corresponding plots or justification are not presented. The authors should include the elbow curve or other validation metrics (e.g., silhouette scores) in the appendix or methods figure to enhance traceability of data processing.

 8. The study uses an 80/20 training-validation split, whereas Molesworth et al. (2003) employed a 60/40 split. The higher AUC (0.908 vs 0.82) may partly reflect this difference rather than a true improvement in model performance. The authors should justify their choice of partition ratio and, ideally, replicate the analysis using a 60/40 split for direct comparability.

**Minor comments**

 9. Only the final retained variables’ cluster maps are shown, while those of excluded variables (wind speed, land-cover) are omitted. Please provide clustering outputs for all variables in the Supplementary Materials to clarify the full environmental data-processing workflow.

 10. Periods are missing at the end of the sections *“Logistic regression model sensitivity and specificity,” “Key findings,”* and *“Limitations and future research.”*

 11. In Figure 2(b), the label should read “specific humidity (g/m³)” rather than “specific humidity (g/m3).” All figures and tables should use standardized SI units and consistent typographical formatting.

Is the work clearly and accurately presented and does it cite the current literature?

Partly

If applicable, is the statistical analysis and its interpretation appropriate?

Partly

Are all the source data underlying the results available to ensure full reproducibility?

Partly

Is the study design appropriate and is the work technically sound?

Partly

Are the conclusions drawn adequately supported by the results?

Partly

Are sufficient details of methods and analysis provided to allow replication by others?

Partly

Reviewer Expertise:

Mainly engaged in research on environment and health, with a focus on the impacts of climate change, air pollution, and other environmental factors on population health, including the environmental drivers of infectious disease transmission.

I confirm that I have read this submission and believe that I have an appropriate level of expertise to confirm that it is of an acceptable scientific standard, however I have significant reservations, as outlined above.

# Response

**Published:** 2026-01-21

**Authors:** Molly Cliff

**Affiliations:** University of Cambridge Department of Veterinary Medicine, Cambridge, England, UK

We would like to thank the reviewer for both taking the time to review this manuscript and for the insightful comments contained within the review. In this response we display the reviewer’s comments in normal text and our response in italics  

**Major comments**

 1. The current title, “Updating the meningitis belt,” implies that the research re-defines the spatial boundaries of the belt. Yet, after excluding the DRC, the distribution of high-risk areas remains largely consistent with Molesworth et al. (2003), without any confirmed expansion. This could mislead readers regarding the novelty of the findings. The authors are encouraged to reconsider the title and reposition the study focus. If no new belt boundary has been empirically defined, alternative titles such as “Re-evaluating environmental associations with meningitis risk across Africa” or “Revalidation of environmental determinants of meningitis outbreaks” would more accurately reflect the study’s scope.

*The study title has been changed to reflect the study’s scope and focus as suggested.*

 2. The introduction cites Chen et al. (2023), who demonstrated a significant association between temperature variability and meningitis incidence, yet temperature was not included in the present model. The authors should clarify whether this omission was due to multicollinearity, data availability, or model stability. Given temperature’s central role in dry-season transmission mechanisms, its exclusion requires explicit justification based on statistical or theoretical grounds.

*Within our paper we have rerun the analysis and reconsidered the role of temperature. Temperature was excluded from the final model due to collinearity with other factors as and our decision to prioritise Aerosol Optical Depth within the analysis.*

 3. The authors deliberately modelled only environmental variables to avoid immunological confounding. However, since the large-scale introduction of MenAfriVac after 2010 has profoundly altered meningitis epidemiology, omitting vaccination coverage, population immunity, migration, and urbanization may overstate the role of environmental predictors. The discussion should explicitly acknowledge this limitation and speculate how inclusion of immunization or socio-demographic variables might alter the observed associations.

*We have updated our analysis to include a sensitivity analysis which only considered outbreaks that occurred prior to the introduction of MenAfriVac on a country level. Details of this analysis can be found in both the results section of the manuscript as well as the extended data. Furthermore, within our discussion we examine how the inclusion of vaccination coverage and socio-demographic variables might alter the observed associations. One of the key challenges of analysing both vaccination coverage and socio-demographic variables is the availability of such data. It is unlikely that such data would be available of continental level at an appropriate resolution for inclusion within the model.*

 4. The DRC substantially affects spatial risk estimates, yet the explanation provided—“limited laboratory confirmation”—is insufficient. The authors should explore surveillance system discrepancies, reporting biases, and potential spatial misclassification (case reporting site vs infection origin). The Methods section should also specify how spatial mismatches and uncertainty were addressed (e.g., overlap thresholds, interpolation precision, temporal alignment).

*Unfortunately, there is limited information and literature regarding surveillance system discrepancies, reporting biases, and spatial misclassification for meningitis outbreaks in the DRC. We have tried to address this information gap by describing a* *2021 outbreak of meningitis within the DRC that highlights issues regarding national surveillance and outbreak reporting* *If the reviewer is aware of data sources or literature that could provide insight into surveillance system discrepancies, reporting biases, and potential spatial misclassification within the DRC we would welcome this guidance as we could then explore these issues further within future analyses.* *Further to this our methods section already describes the handling of spatial uncertainty, including spatial overlap between WHO and GADM ADMN2 shapefiles to address district boundary changes as well as overlap thresholds. As this model is a static outbreak analysis with districts marked as 1/0 if they had ever experienced an outbreak within a 20-year period, temporal uncertainty remains limited. We have clarified the methodology to emphasise these points further.*

 5. The paper’s main contribution lies in: (i) extending the temporal window by 20 years; (ii) using substantially higher-resolution environmental datasets; (iii) maintaining methodological comparability with Molesworth et al. to enable longitudinal evaluation; and (iv) introducing wind-speed as a new variable. These advances should be systematically emphasized in the Introduction and Discussion, with explicit comparisons to Molesworth (2003), Savory (2006), and Tall (2022) to highlight methodological and policy-relevant innovations. *Thank you for this suggestion.*

*Both the introduction and discussion have been revised to reflect the contributions of this paper. We have emphasised the extended temporal window, the use of higher-resolution environmental datasets, methodological comparability with Molesworth et al., and the inclusion of wind speed as a novel variable. Furthermore, we have made explicit comparisons to Molesworth (2003), Savory (2006), and Tall (2022) in both the introduction and the discussion to highlight methodological advancements of this analysis alongside policy-relevant innovations.*

 6. Wind speed and land-cover were excluded from the final model, yet both are ecologically relevant—especially wind speed, which is closely linked to aerosol dispersion during the dry season. The authors should provide detailed information on stepwise-selection criteria (e.g., significance thresholds), multicollinearity diagnostics (e.g., VIF values), and decision rules for exclusion. Supplementary material should include model performance comparisons with and without these variables to improve transparency and reproducibility.

*We have updated the methodology section to include detailed information regarding backwards stepwise analysis to reach the final model, including multicollinearity diagnostics and the decision rules used for variable exclusion. We have also rerun the analysis and updated the model to include windspeed as a variable within the final model.* *Alongside this we have updated the extended information section to include model performance comparisons with and without landcover, highlighting our decision to opt for a more parsimonious model as well as to improve transparency and reproducibility.*

 7. The Methods section notes the use of iterative modeling and the elbow method to determine the optimal number of clusters, but the corresponding plots or justification are not presented. The authors should include the elbow curve or other validation metrics (e.g., silhouette scores) in the appendix or methods figure to enhance traceability of data processing.

*We have updated the methodology section of our paper to include parameter details for clusters, including number of clusters chosen, number of starts and convergence criteria. Alongside this our extended data includes random seeds and elbow plots for all variables, allowing for improved reproducibility. We have rerun the k-means clustering analysis increasing both the number of random starts and iterations from the default settings. In addition,  district level outbreak weighting was subsequently adjusted to optimise sensitivity and specificity.*

 8. The study uses an 80/20 training-validation split, whereas Molesworth et al. (2003) employed a 60/40 split. The higher AUC (0.908 vs 0.82) may partly reflect this difference rather than a true improvement in model performance. The authors should justify their choice of partition ratio and, ideally, replicate the analysis using a 60/40 split for direct comparability.

*Within the methods section we have clarified that we initially chose an 80/20 split as we had a smaller number of outbreaks affected districts as compared to Molesworth et al. (2003). To allow for comparability we have also replicated the analysis using a 60/40 split and found that model sensitivity and specificity remain similar to the results of the 80/20 split*

**Minor comments**

 9. Only the final retained variables’ cluster maps are shown, while those of excluded variables (wind speed, land-cover) are omitted. Please provide clustering outputs for all variables in the Supplementary Materials to clarify the full environmental data-processing workflow.

*Within the methods section we have provided clustering outputs for all temporal variables. As we used static values for landcover, no clustering was carried out on this variable with the static categorical values being used within our model.*

 10. Periods are missing at the end of the sections “Logistic regression model sensitivity and specificity,” “Key findings,” and “Limitations and future research.”

*This has now been corrected*

 11. In Figure 2(b), the label should read “specific humidity (g/m³)” rather than “specific humidity (g/m3).” All figures and tables should use standardized SI units and consistent typographical formatting. *This has now been updated*

# Reviewer report: Reviewer response for version 1

**DOI:** 10.21956/wellcomeopenres.27291.r135924

**Published:** 2025-11-11

**Authors:** Junjun Chen

**Affiliations:** 1 Johns Hopkins University, Baltimore, USA

**Summary**

 The authors present an updated environmental risk map of epidemic bacterial meningitis in Africa (2003 to 2022) using WHO-AFRO surveillance and modern environmental datasets. Monthly climate variables, rainfall, aerosol optical depth (dust), specific humidity, and windspeed, were reduced into seasonal profiles using PCA and k-means clustering, while land cover was treated as static. Logistic regression linked district-level epidemic history (ever vs never) to these climate profiles and land cover types.

 After stepwise selection, the final model retained rainfall, dust, and humidity as key predictors, achieving an AUC of 0.908 with roughly 81% specificity and 84% sensitivity (p > 0.4). Excluding the DRC improved AUC to 0.944 and increased specificity by about 11%. The resulting risk map recovered the canonical Sahelian belt as the highest-risk zone, while the moderate risk signal seen in peripheral countries (Republic of Congo, Gabon, Liberia, Angola) disappeared once the DRC was excluded, highlighting how data quality and laboratory confirmation critically shape risk interpretation. The authors appropriately emphasize improved diagnostics and surveillance capacity under the WHO “Defeating Meningitis by 2030” roadmap.

 This is a strong and timely study that (1) reaffirms the robustness of Sahel-linked climatic drivers of meningitis, (2) demonstrates excellent predictive performance, and (3) responsibly examines the appearance of “belt expansion” through a well-designed sensitivity analysis that attributes it to surveillance bias rather than ecological change. Addressing several methodological and reporting refinements, especially spatial dependence, uncertainty communication, and clustering reproducibility, would further enhance both the scientific rigor and the operational utility of this work.

 I am genuinely excited for this paper’s indexing. It is an important and impactful contribution to climate-epidemiological modeling and public-health policy in sub-Saharan Africa.

**Strengths**

 This manuscript sets a clear comparative objective against the classic Molesworth model, allowing time-consistent inference while leveraging modern, high-resolution environmental data. The authors provide transparent performance metrics and a particularly insightful DRC sensitivity analysis that directly addresses surveillance quality. Moreover, the policy relevance is substantial: the findings have clear implications for early-warning systems, surveillance strengthening, and resource allocation across the meningitis belt.

**Suggestions**

**1. Uncertainty communication**

 Only point probabilities are mapped. For policy-relevant interpretation, users need a sense of uncertainty, especially in peripheral or moderate-risk regions. Consider adding uncertainty layers (eg, standard errors or 95% CIs of predicted probabilities) and/or regional calibration plots to convey confidence in model outputs.

**2. Clustering reproducibility**

 Clarify the k-means clustering setup and reproducibility parameters. While the workflow is outlined, exact specifications, such as the number of clusters per variable, selection criteria (elbow, silhouette, or SSE), initialization method, number of starts, stopping rule, and random seeds, should be reported. This transparency would greatly improve reproducibility.

**3. District mapping sensitivity**

 Potential misclassification may arise when mapping historical outbreaks to 2022 GADM boundaries using 50%/30% overlap thresholds. A short sensitivity analysis varying these thresholds (eg, 40% and 60%) and reporting stability in coefficients or AUC would demonstrate robustness to boundary uncertainty.

**4. Data reproducibility**

 Because the WHO-AFRO outbreak data are not publicly accessible, full replication is currently limited. To improve transparency, consider releasing an anonymized proxy dataset (eg, district-level binary outcomes by country with masked IDs) or a deterministic script that reconstructs the analysis dataset from a standard WHO extract format. This would allow authorized users to fully reproduce the results.

**5. Vaccination and immunity context**

 The exclusion of vaccination variables is reasonable to isolate environmental effects, but a brief sensitivity discussion on whether including simple covariates, such as MenAfriVac rollout year or PCV introduction by country, affects calibration or association strength would strengthen the interpretation.

**6. Recurrent-epidemic districts**

 It would be valuable to include a table of the top 20 districts with recurrent epidemics, along with their corresponding climate classes. This addition would enhance practical relevance for outbreak preparedness programs and country-level health planners.

Is the work clearly and accurately presented and does it cite the current literature?

Yes

If applicable, is the statistical analysis and its interpretation appropriate?

Yes

Are all the source data underlying the results available to ensure full reproducibility?

Yes

Is the study design appropriate and is the work technically sound?

Yes

Are the conclusions drawn adequately supported by the results?

Yes

Are sufficient details of methods and analysis provided to allow replication by others?

Yes

Reviewer Expertise:

Biomedical engineering, public health data science, environmental epidemiology, and AI-driven modeling of disease risk.

I confirm that I have read this submission and believe that I have an appropriate level of expertise to confirm that it is of an acceptable scientific standard, however I have significant reservations, as outlined above.

# Response

**Published:** 2026-01-21

**Authors:** Molly Cliff

**Affiliations:** University of Cambridge Department of Veterinary Medicine, Cambridge, England, UK

We would like to thank the reviewer for both taking the time to review this manuscript and for the insightful comments contained within the review. In this response we display the reviewer’s comments in normal text and our response in italics  

1. **Uncertainty communication**
**Only point probabilities are mapped. For policy-relevant interpretation, users need a sense of uncertainty, especially in peripheral or moderate-risk regions. Consider adding uncertainty layers (eg, standard errors or 95% CIs of predicted probabilities) and/or regional calibration plots to convey confidence in model outputs.**

***To enhance  policy relevant interpretation we have added in risk maps with 95% CI have been added into the extended data, to convey confidence in model outputs.***

**2. Clustering reproducibility**

**Clarify the k-means clustering setup and reproducibility parameters. While the workflow is outlined, exact specifications, such as the number of clusters per variable, selection criteria (elbow, silhouette, or SSE), initialization method, number of starts, stopping rule, and random seeds, should be reported. This transparency would greatly improve reproducibility.** *We have updated the methodology section of our paper to include parameter details for clusters, including number of clusters chosen, number of starts and convergence criteria. Alongside this our extended data includes random seeds and elbow plots for all variables, allowing for improved reproducibility. We have updated the k-means clustering analysis, increasing both the number of random starts and iterations from the default settings. Furthermore, district level outbreak weighting was adjusted to optimise sensitivity and specificity.*

1. **District mapping sensitivity**
  Potential misclassification may arise when mapping historical outbreaks to 2022 GADM boundaries using 50%/30% overlap thresholds. A short sensitivity analysis varying these thresholds (eg, 40% and 60%) and reporting stability in coefficients or AUC would demonstrate robustness to boundary uncertainty.

 
*Within our methodology and results section, we have defined a sensitivity analysis that we undertook varying the overlap threshold of historical outbreaks to 40% and 60%. Within this analysis the AUC remained at 0.908, as only four district outbreaks were affected by the small boundary changes. *  

1. **Data reproducibility**
  Because the WHO-AFRO outbreak data are not publicly accessible, full replication is currently limited. To improve transparency, consider releasing an anonymized proxy dataset (eg, district-level binary outcomes by country with masked IDs) or a deterministic script that reconstructs the analysis dataset from a standard WHO extract format. This would allow authorized users to fully reproduce the results.

*Unfortunately, it remains difficult to release an anonymized proxy version of the raw data, as the spatial identifiers within such a dataset would make it possible to identify the dates and locations of outbreaks. Furthermore, the outbreak dataset from WHO-AFRO is subject to explicit data usage and redistribution restrictions, preventing its release.* *To maximise reproducibility of results all datasets apart from the raw epidemiological surveillance data which is subject to data sharing restrictions from the World Health Organisation are publicly available. All processed datasets are available as part of the underlying data.* *Figshare: Updating the meningitis belt: associations between environmental factors and epidemic meningitis risk across Africa- Supporting data https://doi.org/10.6084/m9.figshare.29900975* *Furthermore, within the manuscript we have documented all processing steps applied to the epidemiological data and made the R code publicly available on GitHub.*

**5. Vaccination and immunity context**

 The exclusion of vaccination variables is reasonable to isolate environmental effects, but a brief sensitivity discussion on whether including simple covariates, such as MenAfriVac rollout year or PCV introduction by country, affects calibration or association strength would strengthen the interpretation.

*We have updated our analysis to include a sensitivity analysis which only considered outbreaks that occurred prior to the introduction of MenAfriVac on a country level. Details of this analysis can be found in both the results section of the manuscript as well as the extended data. Furthermore, within our discussion we examine how the inclusion of vaccination coverage and socio-demographic variables might alter the observed associations.*

**6. Recurrent-epidemic districts**

 It would be valuable to include a table of the top 20 districts with recurrent epidemics, along with their corresponding climate classes. This addition would enhance practical relevance for outbreak preparedness programs and country-level health planners.

*The results section has now been updated to include a table of the top 20 districts with recurrent epidemics, along with their corresponding climate classes*

# Reviewer report: Reviewer response for version 1

**DOI:** 10.21956/wellcomeopenres.27291.r132782

**Published:** 2025-09-30

**Authors:** MUHAMMAD UMAR AHSAN

**Affiliations:** 1 D.G Khan Medical College, Punjab, Pakistan

1. Is the work clearly and accurately presented, and does it cite the current literature? **Partly**
  Some important recent outbreak reports, WHO updates, and vaccination coverage studies are under-cited. 
2. Is the study design appropriate and is the work technically sound? **Partly**
  The decision to exclude vaccination coverage and socio-demographic variables may oversimplify the model. Interpretation of a possible south-westerly expansion may reflect a data artifact. 
3. Are sufficient details of methods and analysis provided to allow replication by others? **Partly **
   Parameter details for clustering (e.g., exact number of clusters chosen, initialization, random seed, convergence criteria) are not fully described.
4.  If applicable, is the statistical analysis and its interpretation appropriate? **I cannot comment**. A qualified statistician is required.
5. Are all the source data underlying the results available to ensure full reproducibility? **No**
  The environmental source data are open, but the epidemiological data and processed datasets are not accessible, which prevents full reproducibility.
6. Are the conclusions drawn adequately supported by the results? **Partly**
  Conclusions are partly supported by the results. The findings regarding rainfall, dust (AOD), and humidity as key environmental drivers, as well as the persistence of the Sahel as the primary high-risk zone, are demonstrated. However, the assertion of a south-westerly expansion of the meningitis belt (into Congo, Gabon, Liberia, and Angola) is less robust. 

Is the work clearly and accurately presented and does it cite the current literature?

Partly

If applicable, is the statistical analysis and its interpretation appropriate?

I cannot comment. A qualified statistician is required.

Are all the source data underlying the results available to ensure full reproducibility?

No

Is the study design appropriate and is the work technically sound?

Partly

Are the conclusions drawn adequately supported by the results?

Partly

Are sufficient details of methods and analysis provided to allow replication by others?

Partly

Reviewer Expertise:

Consequences of climate change on health.

I confirm that I have read this submission and believe that I have an appropriate level of expertise to confirm that it is of an acceptable scientific standard, however I have significant reservations, as outlined above.

# Response

**Published:** 2026-01-21

**Authors:** Molly Cliff

**Affiliations:** University of Cambridge Department of Veterinary Medicine, Cambridge, England, UK

We would like to thank the reviewer for both taking the time to review this manuscript and for the insightful comments contained within the review. In this response we display the reviewer’s comments in normal text and our response in italics

1. Is the work clearly and accurately presented, and does it cite the current literature? **Partly**
  Some important recent outbreak reports, WHO updates, and vaccination coverage studies are under-cited. 

 
*We have updated the manuscript introduction to include details on the introduction of both MenA and Men5CV vaccines across the meningitis belt, citing key studies which also examine vaccination coverage. Additionally, we have included further details of recent bacterial meningitis outbreaks into the paper’s introduction. If the reviewer still feels that we have under cited the literature, we would appreciate details of specific studies or reports that should be included. *  

1. Is the study design appropriate and is the work technically sound? **Partly**
  The decision to exclude vaccination coverage and socio-demographic variables may oversimplify the model. Interpretation of a possible south-westerly expansion may reflect a data artifact. 

*We have updated our analysis to include a sensitivity analysis which only considered outbreaks that occurred prior to the introduction of MenAfriVac on a country level. Details of this analysis can be found in both the results section of the manuscript as well as the extended data. Furthermore, within our discussion we examine how the inclusion of vaccination coverage and socio-demographic variables might alter the observed associations. One of the key challenges of analysing both vaccination coverage and socio-demographic variables is the availability of such data. It is unlikely that such data would be available of continental level at an appropriate resolution for inclusion within the model.* *Regarding the reviewer’s comment that a possible south-westerly expansion may reflect a data artefact, we emphasize that this this a key point of the paper. Our analysis highlights that the possible expansion of the meningitis belt region is likely driven by unconfirmed cases of suspected meningitis  in the **Democratic Republic of the Congo (DRC). Within this paper we argue that the apparent extension of the belt into the Republic of Congo, Gabon and Angola does not represent a climate driven shift in the meningitis belt but is instead likely an artefact of reporting practices in the DRC** **,** with far fewer laboratory confirmed cases relative to neighbouring regions. *

1. Are sufficient details of methods and analysis provided to allow replication by others? **Partly **
   Parameter details for clustering (e.g., exact number of clusters chosen, initialization, random seed, convergence criteria) are not fully described.

*We have updated the methodology section of our paper to include parameter details for clusters, including number of clusters chosen, number of starts and convergence criteria. Alongside this our extended data includes random seeds and elbow plots for all variables, allowing for improved reproducibility. We have rerun the k-means clustering analysis increasing both the number of random starts and iterations from the default settings. In addition, district level outbreak weighting was subsequently adjusted to optimise sensitivity and specificity.*

1.  If applicable, is the statistical analysis and its interpretation appropriate? **I cannot comment**. A qualified statistician is required.
2. Are all the source data underlying the results available to ensure full reproducibility? **No**
  The environmental source data are open, but the epidemiological data and processed datasets are not accessible, which prevents full reproducibility.

*All datasets apart from the raw epidemiological surveillance data which is subject to data sharing restrictions from the World Health Organisation are publicly available. All processed datasets are available as part of the underlying data.* *Figshare: Updating the meningitis belt: associations between environmental factors and epidemic meningitis risk across Africa- Supporting data 10.6084/m9.figshare.29900975).* *Furthermore, within the manuscript we have documented all processing steps applied to the epidemiological data and made the R code publicly available on GitHub. *  

1. Are the conclusions drawn adequately supported by the results? **Partly**
  Conclusions are partly supported by the results. The findings regarding rainfall, dust (AOD), and humidity as key environmental drivers, as well as the persistence of the Sahel as the primary high-risk zone, are demonstrated. However, the assertion of a south-westerly expansion of the meningitis belt (into Congo, Gabon, Liberia, and Angola) is less robust. 

*Regarding the south-westerly expansion of the meningitis belt, as mentioned above this paper argues that this is likely an artefact of reporting practices within the Democratic of Congo. **Within the paper conclusion we advocate for the improvement of** epidemic reporting and diagnostic capacity within meningitis outbreaks, to accurately assess disease risk across the continent.*
